# Electromagnetic Interference Effect of Portable Electronic Device with Satellite Communication to GPS Antenna

**DOI:** 10.3390/s25144438

**Published:** 2025-07-16

**Authors:** Zhenyang Ma, Sijia Zhang, Zhaobin Duan, Yicheng Li

**Affiliations:** 1 Institute of Science and Technology Innovation, Civil Aviation University of China, Tianjin 300300, China; zbduan@cauc.edu.cn; 2 Key Laboratory of Civil Aviation Aircraft Airworthiness Certification Technology, Civil Aviation University of China, Tianjin 300300, China; ycli@cauc.edu.cn; 3 Sino-European Institute of Aviation Engineering, Civil Aviation University of China, Tianjin 300300, China; 2023122041@cauc.edu.cn; 4 College of Safety Science and Engineering, Civil Aviation University of China, Tianjin 300300, China

**Keywords:** portable electronic device, interference path loss, front door coupling, electromagnetic interference

## Abstract

Recent technological advancements have resulted in the emergence of portable electronic devices (PEDs), including mobile phones equipped with satellite communication capabilities. These devices generally emit higher power, which can potentially cause electromagnetic interference to GPS antennas. This study uses both simulation and experimental methods to evaluate the interference path loss (*IPL*) between PEDs located inside an A320 aircraft and an external GPS antenna. The effects of PED location, antenna polarization, and frequency bands on *IPL* were simulated and analyzed. Additionally, measurement experiments were conducted on an A320 aircraft, and statistical methods were used to compare the experimental data with the simulation results. Considering the front-door coupling of both spurious and intentional radiated emissions, the measured *IPL* is up to 15 ± 3 dB lower than the $IPL_{target}$. This result should be interpreted with caution. This issue offers new insights into the potential risks of electromagnetic interference in aviation environments. The findings help quantify the probability of interference with GPS antennas. Furthermore, the modeling simplification method used in this study may be applicable to the analysis of other large and complex structures.

## 1. Introduction

It is common for passengers to carry portable electronic devices (PEDs), such as mobile phones, laptops and game consoles onto aircraft. This trend is due to the fact that the radiation emitted by these wireless devices is not subject to the electromagnetic compatibility standards of airborne equipment [1]. Although the frequency bands used by aircraft navigation systems differ from those used by PEDs, the bands are relatively close. As a result, these devices can cause cross-modulation interference or out-of-band spurious emissions [2]. The complex electromagnetic environment inside the aircraft, the high sensitivity of the onboard navigation antennas, and the wide frequency range of various cabin devices can lead to unavoidable electromagnetic interference [3].

In 2022, the Radio Technical Commission for Aeronautics (RTCA) revised the DO-307B aircraft design and certification document [4]. This document analyzed spurious radiation from various PEDs and defined interference path loss (*IPL*). *IPL* quantifies the energy loss from PEDs to the onboard antenna, with energy primarily coupled through openings such as windows and doors. The document also defines front-door coupling of both spurious and intentional radiated emissions. To assess spurious radiation, measurements are usually carried out using reverberation chambers [5,6], which are complex and costly.

Many theoretical studies have investigated the coupling mechanisms in open-cavity structures [7,8]. However, these theoretical methods are usually only applicable to cavities with regular geometric structures and are difficult to use in complex cabin environments. In engineering practice, many studies focus on *IPL* to assess electromagnetic interference from PEDs in aircraft navigation systems. For example, Shunichi Futatsumori [9] and colleagues measured *IPL* in small general aviation aircraft in the 4 GHz wireless avionics communication band. Fatih Ustuner et al. [10] conducted PED interference tests on ILS and VOR receivers and compared the results with those from scaled models to determine the interference threshold under extreme conditions. However, the results did not reveal the differences between the scaled models and the actual aircraft.

Commercial electromagnetic field simulation software, such as CST, FEKO, and HFSS, are hig y efficient and can reduce the cost of measuring *IPL*. Nicole L. Armstrong et al. [11] used CST to develop an electromagnetic model of the A319, including the dissipative medium inside the cabin, and simulated the coupling effects of an 800 MHz wireless network. Takashi Hikage et al. [12] created a model of the Boeing 777, using FDTD technology and parallel computing to accurately estimate electromagnetic field distributions. However, these studies only considered the statistical characteristics of *IPL* and did not examine interference to onboard antennas. In addition, some researchers [13,14,15] have used machine learning and neural network methods to predict *IPL*. However, these methods still require large amounts of experimental and simulation data, and their accuracy remains limited. Although some studies indicate that the likelihood of PED radiation causing electromagnetic interference during flight is relatively low, the PEDs examined in these studies, such as mobile phones, Bluetooth devices and ultra-wideband devices, usually have low transmit power.

With the rapid advancement of 5G technology, the International Civil Aviation Organization (ICAO) released Circular 360 [16] in 2024, offering guidance on measures to protect radio altimeters from potential harmful interference. The circular addresses the risks of electromagnetic interference when C-band antennas coexist with 5G signals, with a particular emphasis on aviation safety. Some researchers have studied the *IPL* of small-sized fixed-wing aircraft within the radio altimeter frequency band [17]. Recent studies have also extensively investigated the effects of 5G electromagnetic interference on radio altimeters, as well as related protective measures [18,19,20,21]. Meanwhile, many manufacturers have begun integrating satellite communication technology that operates without the need for base stations [22]. For example, a mobile phone with satellite communication capability can transmit at a peak power of up to 2 W (33 dBm) in the 1980–2010 MHz frequency band, which is higher than previous models. This increase in transmit power has raised concerns about possible electromagnetic interference with GNSS systems, including GPS, Galileo, and SBAS.

The coexistence of mobile phones with satellite communication capabilities and aircraft GNSS systems has become a significant concern, especially with respect to modern GNSS frequencies such as GPS L1, Galileo E1/E5, and SBAS L5. The operating frequency band of GPS L1 is close to that used by mobile phone satellite communication systems. Currently, there is no standard for assessing whether mobile phone satellite communication functions can interfere with GPS antennas, and research on this topic is still limited. Although this study specifically focuses on the susceptibility of GPS L1, the results may also provide useful insights for analyzing interference with Galileo E1/E5 and SBAS L5. This paper presents the basic principles of electromagnetic compatibility assessment by analyzing the *IPL* loss mechanism. CST(2016) is used to construct an A320 cabin model for *IPL* calculation, and measurement experiments are conducted to validate the model and simulation approach. The purpose of this study is to explore the potential electromagnetic interference risks posed by these devices and to propose methods to evaluate their impact on GPS antennas.

## 2. Basic Principles

*IPL* serves as a key indicator for evaluating electromagnetic interference defined in Equation (1):

(1) $$IPL=P_{T}-P_{R}$$

where $P_{T}$ denotes the transmit power and $P_{R}$ denotes the received power (in dBm). *IPL* can be equivalently expressed as the scattering parameter $S_{21}$ between the antenna ports in Equation (2):

(2) $$IPL=|S_{21}|=\frac{|V_{2}|}{|V_{1}|}=20\log\left(\frac{|V_{2}|}{|V_{1}|}\right)$$

Note that the magnitude $|S_{21}|$ is used in this equation, as the negative sign in $S_{21}$ indicates the signal direction, while the positive magnitude represents the loss. This approach ensures that negative $S_{21}$ values are treated as positive losses in the calculation. Additionally, the use of the 20log form is appropriate because CST exports $S_{21}$ as a voltage-wave ratio, which requires logarithmic conversion to express the result in decibels. To assess whether high power PEDs can cause electromagnetic interference to the onboard antenna, the $IPL_{target}$ is defined in Equation (3):

(3) $$IPL_{target}=P_{T}+MEF-P_{threshold}$$

where $P_{threshold}$ denotes the interference threshold of the receiving antenna and MEF denotes the front door multiple equipment factor. To compute the cumulative effects from multiple devices, the spurious emission value for each device is first weighted proportionally to its linear interference coupling value. The results for all devices are then summed and normalized to the single PED worst-case contribution to arrive at the cumulative effects. When the sources have equal magnitude, the worst-case contribution from a single PED corresponds to the location with the lowest *IPL* value. The interference coupling value $C_{i}$ is calculated from the *IPL* at the same source location, as described in [23]:

(4) $$C_{i}=10^{-IPL/10}$$

Thus, the maximum power coupled from seat *n* to the receiving antenna can be expressed as follows:

(5) $$P_{R}^{n}=P_{T}^{n}\cdot C_{i}^{n}$$

By summing all $P_{R}^{n}$ and normalizing by the maximum value $P_{R}^{\max}$, the MEF for *N* devices is defined as follows:

(6) $$MEF=\left(\sum_{N}P_{R}^{n}\right)/{\left(P_{R}^{n}\right)}^{\max}=\left(\sum_{N}P_{T}^{n}\cdot C_{i}^{n}\right)/{\left(P_{T}^{n}\cdot C_{i}^{n}\right)}^{\max}$$

If $P_{T}$ is identical for all transmitting sources, it can be normalized out and Equation (6) becomes

(7) $$MEF=\sum_{N}\left(C_{i}^{n}/C_{i}^{\max}\right);n=1,\ldots ,N$$

Alternatively, the normalized coupling factor $\langle C_{i}^{n}\rangle$ and the normalized *IPL* $\langle IPL^{n}\rangle$ can be defined as follows:

(8) $$\begin{array}{cc} \langle IPL^{n}\rangle & =IPL^{n}-IPL^{\min},\;\mathrm{and}\\ \langle C_{i}^{n}\rangle & =C_{i}^{n}/C_{i}^{\max}=10^{-\langle IPL^{n}\rangle /10} \end{array}$$

It can be shown that

(9) $${MEF}_{\mathrm{dB}}=10\log_{10}\left(MEF\right)=10\log_{10}\left(\sum_{N}\langle C_{i}^{n}\rangle \right)$$

If the *IPL* is lower than $IPL_{target}$, it means the energy received by the antenna exceeds the interference threshold, which may result in electromagnetic interference to the GPS antenna. Conversely, if the *IPL* is greater than $IPL_{target}$, interference with the antenna is unlikely. For example, a certain type of mobile phone connected to the TianTong satellite operates with an uplink frequency of 1980–2010 MHz and a downlink frequency of 2170–2200 MHz. When the satellite communication function is activated, the device transmits at a peak power of 2 W within this frequency band, which is close to the GPS L1 band. When the satellite communication function is activated, the device transmits at a peak power of 2 W within this frequency band, which is close to the GPS L1 band. The GPS antenna operates at 1575.42 ± 10 MHz, with an interference threshold $P_{threshold}$ of −120.5 dBm, as specified by ICAO Annex 10 [24].

Sensitive receivers can be disrupted by spurious emissions, which may overlap with the receiver’s operating band and significantly degrade performance. In NASA’s research [25], a spectrum analyzer was used to measure the spurious radiation power of various 3G mobile phones in the 1565–1585 MHz range, and the results were compared with the noise floor. The results showed that the spurious radiation power did not exceed −50 dBm. However, mobile phones that connect to the TianTong satellite operate in a frequency band closer to the GPS L1 band and are new products introduced in the past three years. No measurements of their spurious radiation power have been reported. Therefore, NASA’s measurement data can only be used as a reference for this study. This study does not measure the spurious radiation from this type of mobile phone, as the exact value is not the main focus of this research. Instead, the relevant standards [26] are referenced, which specify a spurious radiation limit of −40 dBm in the GPS antenna’s operating frequency band. Therefore, the emission limit specified in the standard is likely based on a worst-case scenario. Using the spurious radiation power and the MEF, the $IPL_{target}$ can be determined.

Additionally, the transmitting antenna was configured to emit a 5G NR TM1.1 standard waveform during the experiment. The 5G NR TM1.1 standard waveform is defined by the Third Generation Partnership Project (3GPP), ensuring that this study aligns with current standards [27]. To determine whether this device may cause electromagnetic interference with the GPS antenna, the *IPL* value should be calculated and compared with $IPL_{target}$.

## 3. A320 Cabin Interference Path Loss Simulation

### 3.1. Model Design and Simulation Setting

**Figure 1:**
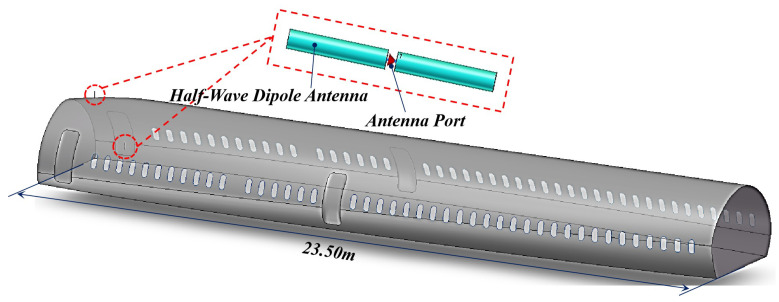
A320 aircraft cabin model.

This research constructs a 1:1 simulation model of the A320 aircraft, as shown in Figure 1. In order to simplify the model, the cockpit, wings, cargo, and other external structures were omitted during the model design process. This approach not only simplifies the model but also reduces the number of mesh cells and shortens the simulation time.

**Figure 2:**
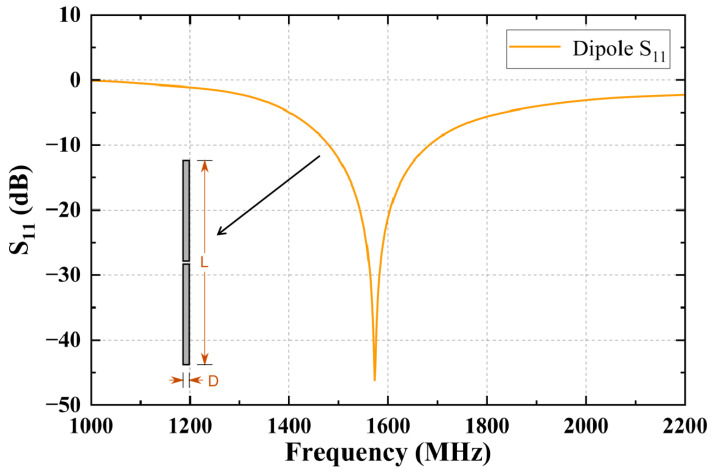
$S_{11}$ of half-wave dipole antenna.

The A320 cabin model is 23.50 m in length and has a fuselage width of 3.95 m. The aircraft is equipped with windows, main cabin doors, and emergency exits on both sides of the fuselage. Both the PED and the GPS antenna are modeled as half-wave dipole antennas to simulate the emission source. The GPS antenna is positioned on the upper surface of the aircraft near the front cabin door. Discrete ports are assigned to both the PED and GPS antennas, as shown in Figure 1. The half-wave dipole antenna used in this study has a length of L = 86.28 mm and a diameter of D = 0.75 mm. The center frequency is set to 1.575 GHz, which matches the operating frequency of the GPS antenna. The gap between the two dipole conductors is set to L/200, and the simulated $S_{11}$ parameter is shown in Figure 2. It is important to ensure that the operating frequency range of the dipole antenna covers the simulation frequency range of 1500–1600 MHz described in the next section. Similarly, if the simulation frequency band is changed, the length of the dipole antenna should be adjusted according to the new center frequency.

**Figure 3:**
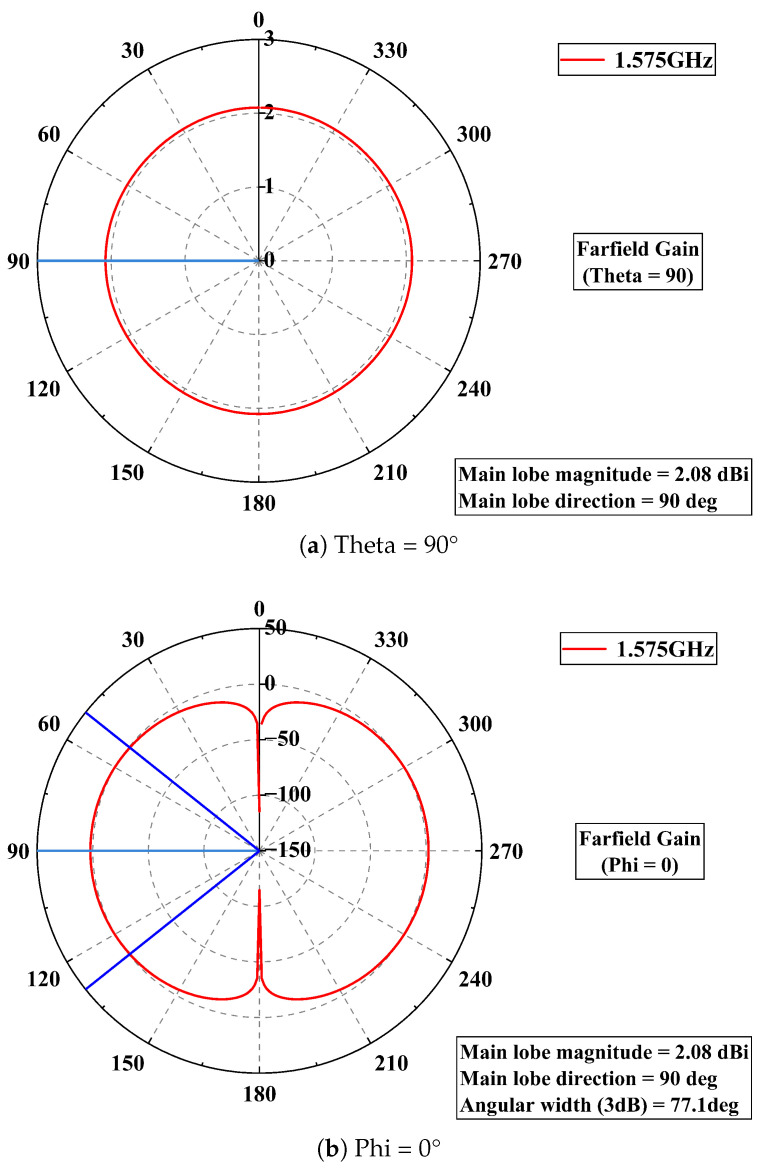
Radiation patterns of antenna. (red line: Farfield Gain; light blue line: Main lobe direction; dark blue: 3dB Angular width).

Furthermore, Figure 3 shows the radiation patterns of the half-wave dipole antenna used in this study on the two principal planes (0 and 90°). When the frequency is set to 1.575 GHz, the antenna’s main lobe gain is 2.08 dBi, and the main lobe is directed at 90°.

Studies [11] have shown that when the aircraft fuselage is modeled as a perfect electric conductor (PEC) with open windows, and another model uses an air fuselage with dielectric windows, the simulation results are similar. Although using a finer mesh can improve accuracy, an excessively fine mesh greatly increases the number of mesh cells and lengthens the simulation time. Studies [11] have also shown that when the mesh size is smaller than λ/8, further refinement has little effect on accuracy but significantly increases the simulation time. The mesh settings used in the simulation are listed in Table 1.

In this study, the structure is configured as a “PEC fuselage with open windows”. The entire model is constructed as a “shell” model in CST, with all six boundaries of the bounding box set to “open (add space)”. The accuracy parameter for the time domain solver is set to −40 dB, which serves as a relative energy threshold commonly used to determine convergence in full-wave electromagnetic simulations. This value is not an absolute power unit (such as dBm), but a normalized criterion based on the system’s initial excitation.

### 3.2. Simulation Results and Analysis

**Figure 4:**
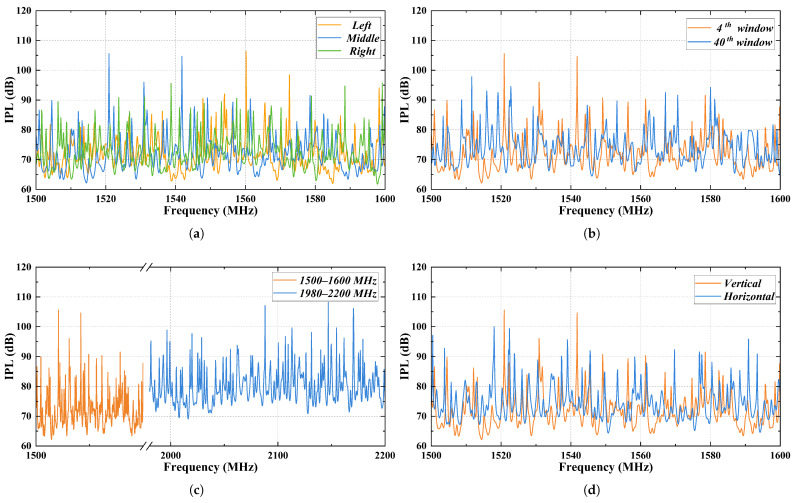
Simulated analysis of factors influencing *IPL*: (**a**) PED at the left, center and right side of the 4th row of windows. (**b**) PED at the 4th and 40th row of windows. (**c**) PED at different frequency bands. (**d**) PED at the 4th row of windows with different polarization methods.

The aircraft cabin has a total of 40 rows of windows. A dipole antenna is placed at each window position in the cabin, and the $S_{21}$ parameter is calculated for each location. According to Equation (2), $S_{21}$ represents the *IPL* value. For each window position, the dipole antenna sweeps the frequency range and the *IPL* value is determined. Since the mobile phone supports the Tiantong satellite communication system, which operates in the 1980–2010 MHz and 2170–2200 MHz frequency bands, this study presents simulated *IPL* values between the GPS antenna and a high power PED in the 1500–1600 MHz and 1980–2200 MHz bands. During the simulation, 1000 sample points are collected within each frequency range. Various factors, including the PED position within the same row of windows, positions across different rows, different frequency bands, and antenna polarization methods, are analyzed to assess their impact on the *IPL*. The results are shown in Figure 4.

To avoid presenting an excessive amount of simulation data, this paper shows only a selection of results to analyze the variation tendency of *IPL* values. The analysis focuses on the maximum, minimum and average *IPL* values under various influencing factors, and calculates the standard deviation (SD), and 95% confidence interval (CI). A statistical analysis of the results is provided in Table 2.

The confidence interval shown in Table 2 indicates a 95% probability that the average *IPL* value falls within this range. When the PED is positioned at different locations (left, center and right) within the same row of windows, the *IPL* values are 71.36 ± 0.32, 71.81 ± 0.35, and 71.60 ± 0.33 dB, respectively. A significance test was performed on the simulation results for the different conditions listed in the table. The results indicate that there is no significant difference when the PED is located at the left, center, or right side of the windows. Therefore, only the case with the PED placed on the right side of the cabin is considered in subsequent simulations. When the PED is moved to the 40th row of windows, the average *IPL* value increases to 75.86 ± 0.32 dB, which is 4.26 dB higher than at the 4th row. This increase is primarily due to the GPS antenna’s location, and as the distance inside the cabin increases, the *IPL* value rises.

Each dipole antenna was vertically polarized in the above research. When the polarization of the dipole antenna inside the cabin is changed, while keeping the frequency band at 1500–1600 MHz, the average *IPL* increases to 74.25 ± 0.33 dB, which is 2.44 dB higher than the value for vertical polarization at the same location. This increase is likely because the vertical polarization aligns with the cabin doors and other apertures, enhancing the electromagnetic coupling. As a result, more energy is received by the external antenna, reducing the average *IPL* value. When the dipole antenna is set to vertical polarization and the frequency band is changed from 1500 to 1600 MHz to the satellite communication band of 1980–2200 MHz, the average *IPL* rises to 79.88 ± 0.35 dB, which is 8.28 dB higher than in the 1500–1600 MHz band. This indicates that increasing the frequency significantly increases the *IPL* value.

Except for the comparison of PED placement at different positions within the same row of windows, all other comparisons showed significant differences according to the significance test. This indicates that the trends observed in the simulation are reliable. To further validate the simulation results, field measurement experiments are conducted in the next chapter.

## 4. A320 Cabin Interference Path Loss Measurement

### 4.1. Experiment Preparation

**Figure 5:**
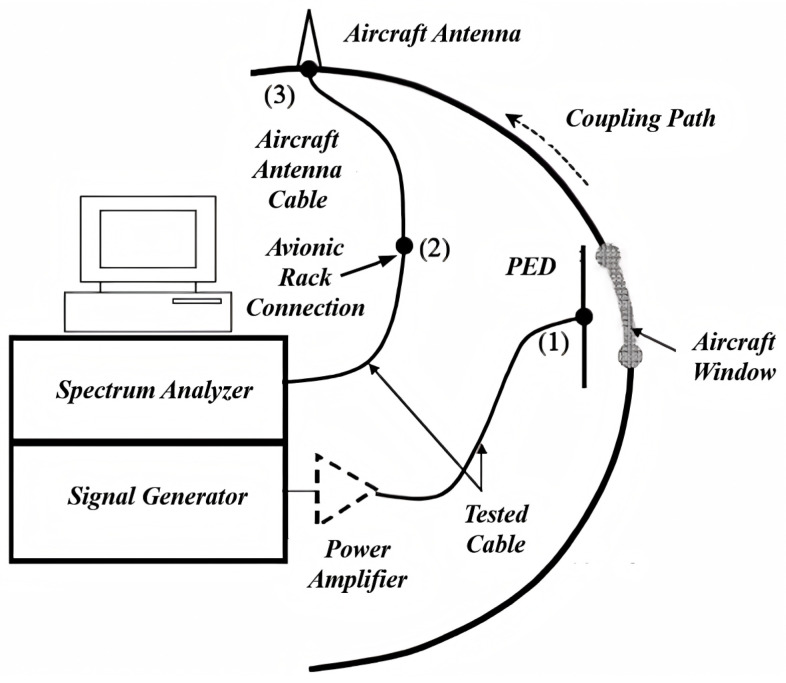
Schematic of the experiment.

The measurement experiment was conducted on an A320-232 aircraft, which has a fuselage width of 3.95 m and a length of 37.57 m. The aircraft is equipped with 40 rows of windows and the positions of these windows align with the simulation model shown in Figure 1. In the simulation, the magnitude of $S_{21}$ between the two antenna ports is used as the *IPL* value. However, it is not feasible to directly measure the $S_{21}$ parameter using a vector network analyzer, due to experimental limitations and other factors. Instead, *IPL* is measured as the difference between the received power $P_{R}$ at the GPS antenna and the transmit power $P_{T}$ of the PED. The schematic diagram of the experimental setup is shown in Figure 5.

**Figure 6:**
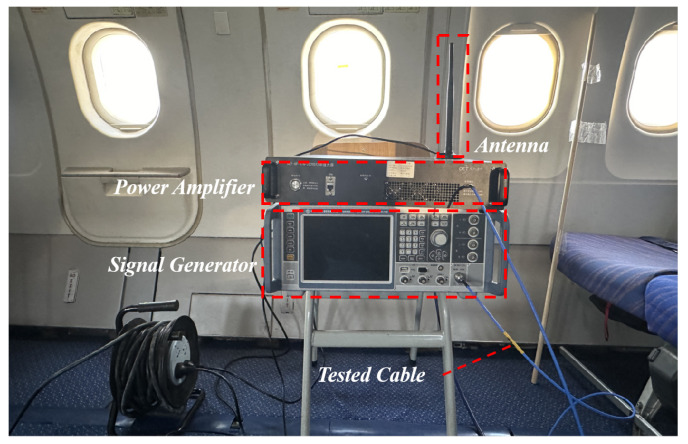
Signal transmission setup.

**Figure 7:**
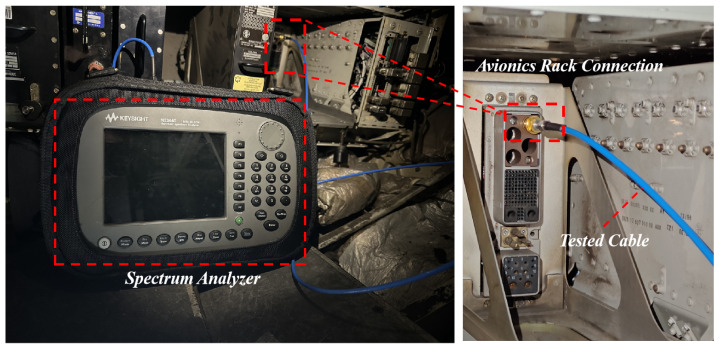
Signal reception setup.

The experimental setup consists of two main components: (1) A signal generator is connected to a power amplifier, which supplies RF power to the PED. (2) A spectrum analyzer is connected to the “avionics rack connection” through tested cables or directly measuring the received power near the aircraft antenna. If the test cables could be connected directly to the aircraft antenna, the measurement results would be more accurate. However, due to the location constraints of the GPS antenna, the tested cables can only be connected to the “avionics rack connection”, which inevitably introduces some cable losses. The actual measurement setup used in the experiment is shown in Figure 6 and Figure 7.

The vector signal generator (ROHDE SCHWARZ SMW-200A, ROHDE SCHWARZ, Munich, Germany) was connected to a power amplifier (OET-GF-1-6-20200, OET, Beijing, China). The output of the amplifier was then connected to an antenna, simulating the use of PED inside the aircraft cabin, as shown Figure 6. It was difficult to directly measure the received power near the GPS antenna, since it is located on the upper surface of the A320 aircraft. The GPS antenna is actually connected to the GLU-920 multi-mode navigation receiver in the forward electrical equipment bay via internal antenna cables. To facilitate measurement, the receiver was removed and a high-shielding effectiveness tested cable was connected at the “avionics rack connection” in order to measure the received power of the GPS antenna. The other end of the tested cable was connected to the spectrum analyzer as shown in Figure 7. The position of PED was varied from front to back within the cabin, positioning it at the left or right side of the same row of windows to assess its effect on the received power.

### 4.2. Experiment Results and Analyses

The signal generator was configured to emit a 5G NR TM1.1 standard waveform with a center frequency of 1.995 GHz and a bandwidth of 20 MHz, simulating the satellite communication frequency band used by the mobile phone. According to the datasheet, the amplifier is specified to have a maximum gain variation of ±1.5 dB under a 0 dBm input and a 50 Ω load. To verify gain flatness across the relevant frequency band, measurements were taken at three representative frequencies: 1980 MHz, 2000 MHz, and 2020 MHz. The amplifier gain was configured to 46 dB using the control software. The measured output gains at the three frequencies were 53.74 dB, 53.87 dB, and 53.78 dB, respectively. These measurements reflect the total system gain, including contributions from the amplifier, cable and connector losses. The gain variation across the three frequencies was within ±0.065 dB, and the 95% confidence interval confirms excellent gain flatness and consistency. These results confirm that the amplifier performs as expected and does not introduce any significant frequency-dependent bias into the measurements.

The total transmission power $P_{total}$ was set to 10 W to prevent the received power from being too low, and the spectrum analyzer’s resolution bandwidth (*RBW*) was set to 1 kHz. All the measurements in this study were conducted with a fixed *RBW* of 1 kHz. Using the same *RBW* for all measurements ensures that power readings are comparable and reduces systematic bias. Under these measurement conditions, a statistical analysis was performed on the noise floor of the spectrum analyzer. The calculated mean, standard deviation, and 95% confidence interval are as follows: −105.31 dBm, 0.85 dB, and ±0.12 dBm, respectively. This indicates that the spectrum analyzer maintains good stability in its noise floor.

Since the power measured by the spectrum analyzer corresponds to the energy within the 1 kHz resolution bandwidth, the energy value *x* of the transmitted signal in this bandwidth needs to be calculated using Equation (10):

(10) $$x=10\log\left(\frac{P_{total}}{1mW}\right)-10\log\left(BW\right)+10\log\left(RBW\right)$$

**Figure 8:**
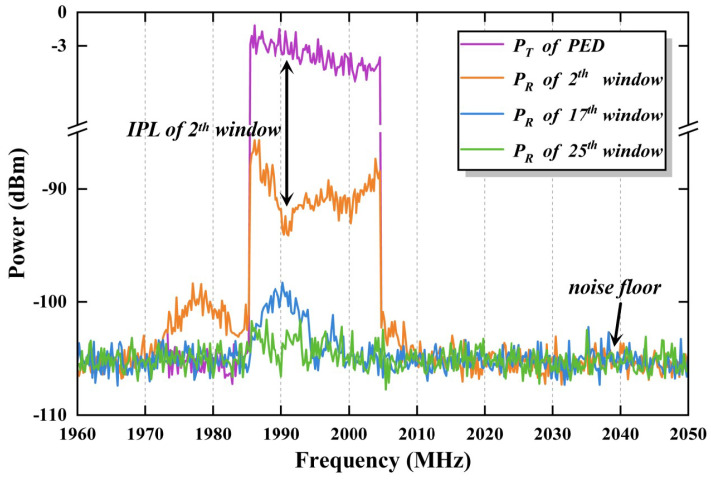
PED emission waveform and measurement results.

By substituting the relevant values into the equation, the transmit power is calculated to be approximately −3 dBm. The difference between the transmit power and the received power represents the *IPL* value. When the transmitting antenna is positioned at the 2nd, 17th and 25th row of windows, the transmit power of the PED and the measurement results from the spectrum analyzer are shown in Figure 8.

The cable loss between the spectrum analyzer and the aircraft antenna must be taken into account in the above calculations. The loss of the blue tested cable shown in Figure 7 was measured to be approximately 2 dB. The cable is connected via an adapter to an ARINC 600 SIZE 2 coaxial pin, which interfaces with the “avionics rack connection”. According to product specifications, the insertion loss introduced by these two connectors is 2 dB. The connection between the avionics rack and the GPS antenna is established using internal aircraft cable. Similar setups have been used in previous NASA measurement experiments [28], where the cable loss was not directly estimated. Based on the previously measured 2 dB loss of the blue tested cable, an estimated loss of 5 ± 2 dB for the internal aircraft cable is considered acceptable. Therefore, the total loss from the GPS antenna to the spectrum analyzer is around 9 ± 2 dB, which is considered a reasonable approximation.

When the transmitting antenna was positioned at the left or right side of the fourth window, the average *IPL* values were determined to be 77.52 dB and 77.15 dB, respectively. This indicates that the placement of PED on either the left or right side of the cabin has a minimal effect on the *IPL* value. Subsequently, the transmitting antenna was positioned at the right side of the cabin, with its location progressively changing from the front to the back of the cabin. The frequency band was set in the GPS antenna’s operating frequency range, or 1985–2005 MHz. Average *IPL* values at various window positions were calculated and compared with the simulation results.

**Figure 9:**
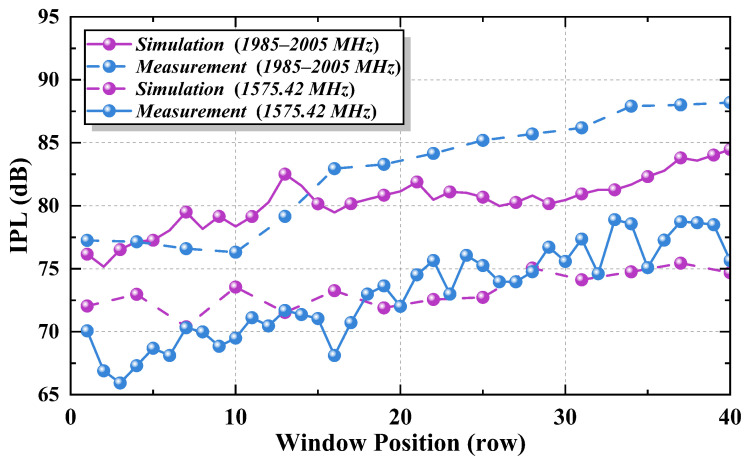
Comparison of *IPL* simulation and experimental results.

These data in Figure 9 demonstrate that the position of the PED influences the *IPL* value. As the distance between the PED and GPS antenna increases, the *IPL* shows a gradual increase. Additionally, a comparison of *IPL* values for two different frequency bands reveals that for the 1575.42 MHz frequency, the *IPL* values range from 65 to 80 dB. In contrast, for the 1985–2005 MHz band, the *IPL* values range from 75 to 90 dB. This indicates that an increase in the measurement frequency corresponds to a noticeable rise in *IPL* values.

**Figure 10:**
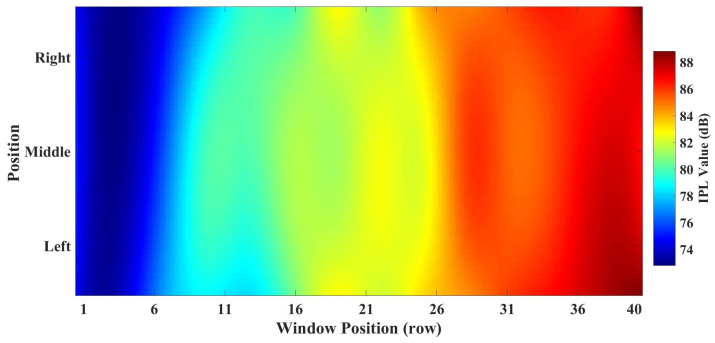
*IPL* value cloud map.

Figure 10 illustrates the *IPL* value cloud map derived from measurement data within the 1985–2005 MHz frequency band. It provides a more intuitive visualization of the variation tendency in *IPL* values.

**Figure 11:**
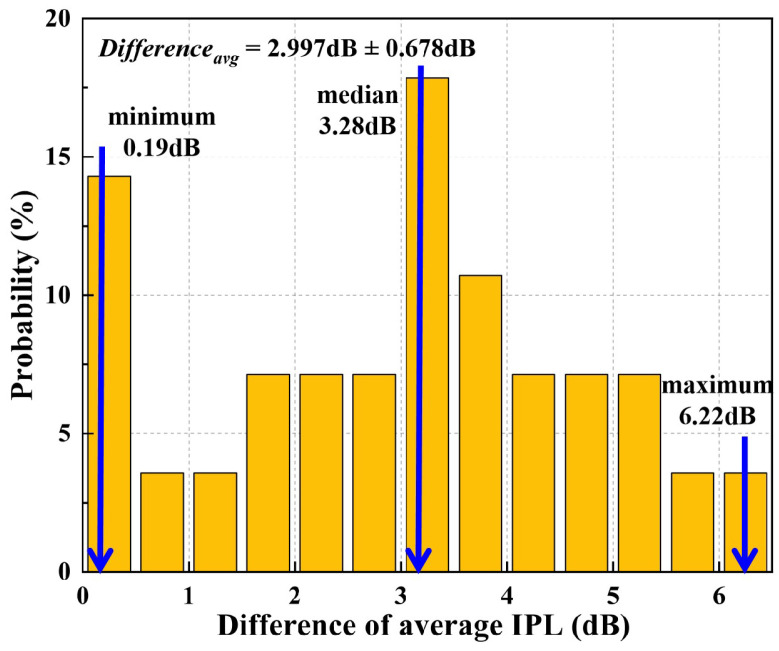
Difference in average *IPL* value between simulation and measurement.

Several factors contribute to the discrepancy between simulation and experiment, including simplifications made in the aircraft model, such as the exclusion of seats, passengers, and luggage racks. Based on the power balance theory, some researchers [29] introduced the quality factor and mean absorption cross-section to quantify the impact of seats and passengers on “power escape through windows”. Variations in aircraft type and the number of seats can also influence the results. The study found that under unoccupied aircraft conditions, lossy media such as seats can reduce the power received outside the window by approximately 5–7 dB. In the experimental setup, the estimated loss from the internal aircraft cable may introduce some uncertainty into the results. Therefore, a statistical analysis was conducted on the difference in average *IPL* values between the simulation and the measurement results, as shown in Figure 11.

To further validate the consistency between the simulation and experimental results, a statistical analysis was conducted on the average *IPL* differences. As shown in Figure 11, the mean difference was 2.997 ± 0.678 dB, with a median of 3.28 dB, a maximum of 6.22 dB, and a minimum of 0.19 dB. The distribution indicates that most deviations are concentrated around 3 dB, with minimal variation. Although a discrepancy exists between the *IPL* values obtained from the simulation and the experimental measurements, the error is considered acceptable. These results suggest that the model and simulation methods used to calculate *IPL* are both valid and feasible, thus supporting the reliability of the methodology in this study.

## 5. Analysis of Front Door Coupling Interference

**Figure 12:**
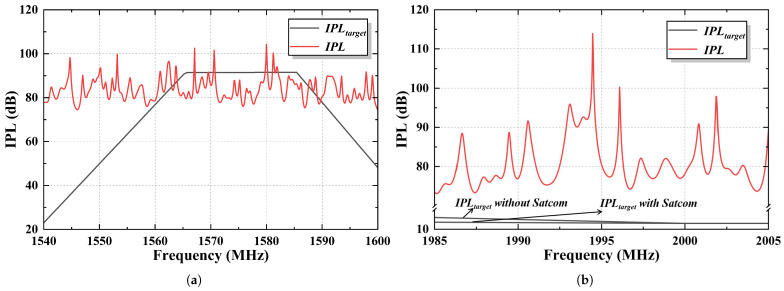
Comparison of *IPL* and $IPL_{target}$: (**a**) NIRA. (**b**) IRA.

The experimental results have confirmed the feasibility of using this model for simulation analysis. Building on the simulation results, this section explores the potential impact of front-door coupling interference. Front-door coupling of spurious radiated emissions (NIRA) refers to the spurious emissions from PEDs that are received by the aircraft’s radio receiver antennas, potentially interfering with the aircraft’s radio receivers. Front-door coupling of intentional radiated emissions (IRA) refers to the intentional emissions from PEDs occurring within licensed frequency bands (such as bands allocated to mobile phones). Considering both types of front-door coupling, if a PED can transmit a 2 W signal within the 1985–2005 MHz frequency range, the $IPL_{target}$ can be calculated. The simulation results are then analyzed to evaluate the electromagnetic interference from the PED to the GPS antenna, comparing *IPL* with $IPL_{target}$, as shown in Figure 12.

It is crucial to hig ight that all results in this study were derived under the worst-case scenario, where the spurious radiation limit was set at −40 dBm. It is certain that the actual measured values will be lower than this threshold. The value of spurious emissions affects the determination of the $IPL_{target}$. If the spurious emission level is lower than −40 dBm, the corresponding $IPL_{target}$ for the NIRA will decrease proportionally.

A comparison of the $IPL_{target}$ with the simulated *IPL* values reveals that for NIRA, the *IPL* does not meet the required interference path loss target at specific frequencies. This indicates that the energy received by the GPS antenna surpasses the interference threshold. For these frequencies, considering the difference of *IPL* between simulation and measurement, the maximum difference between the *IPL* and $IPL_{target}$ is approximately 15 ± 3 dB. A study [10] suggests that when the *IPL* exceeds the $IPL_{target}$ by less than 10 dB, the likelihood of interference is minimal and can be considered negligible. However, the results shown in NIRA should be interpreted with caution. In the case of IRA, the *IPL* far exceeds the $IPL_{target}$ by around 70 dB, suggesting that the intentional emissions from the PED are unlikely to cause interference with the GPS antenna.

All data on GPS interference thresholds are derived from the guidelines in Appendix B, Volume I of ICAO Annex 10 [24]. In the worst-case scenario, spurious emissions reach −40 dBm in the GPS L1 band. We observed a difference of 2.997 ± 0.678 dB between the simulated and measured average *IPL* values. Therefore, a conservative estimate suggests that the spurious emission power of mobile phones must be below −60 dBm in the GPS L1 band when using the satellite communication function to effectively reduce the risk of interference.

In conclusion, during critical flight phases, strict regulation of passengers’ use of satellite communication functions is crucial to prevent interference with the aircraft’s navigation systems. In addition to restricting its use, improving the *IPL* value is vital for minimizing its impact. Research indicates that designing aircraft windows with varying structures and materials can help mitigate the impact of internal electronic devices on antennas [30]. These approaches can be integrated into electromagnetic shielding designs to address more complex operational scenarios.

## 6. Conclusions

This paper investigates the electromagnetic interference impact of high-power PED with satellite communication function on the GPS L1 band. Compared with recent articles, there has been minimal research on the electromagnetic interference effects of satellite communication devices on GPS antennas. This paper may provide some insights for future research in this area. Meanwhile, to face the challenge of large-scale electromagnetic simulations, the model used in this paper omits certain details without altering the main structure of the cabin in order to improve simulation speed. The modeling simplification approach employed could be of potential use in modeling other large, complex structures.

Based on the A320 cabin model with no seats, various measurement positions were selected, and the *IPL* values were simulated across the GPS antennas and satellite communication devices’ frequency range. The results indicate that the placement of PED within the same row of windows has minimal impact on the *IPL*, and variations are mainly determined by the distance between PED and the GPS antenna. The study also demonstrates that when the transmitting antenna is horizontally polarized, the average *IPL* value is significantly higher than when it is vertically polarized. This can be attributed to the fact that the vertical polarization waveform aligns more closely with the cabin doors and apertures, thus enhancing the electromagnetic coupling effects. Additionally, the *IPL* changes for two different frequency bands at the same position reveal that increasing the frequency results in a considerable increase in *IPL*, which reduces the electromagnetic coupling effect.

To validate the simulation results, an experimental measurement setup was designed. A spectrum analyzer was used to measure the received power at the GPS antenna, and the *IPL* value was calculated by comparing the difference between the received power and the transmit power. Experiments were carried out using an A320 aircraft model (Airbus, Toulouse, France), and the results were compared with the simulation data, showing strong consistency. Considering the operating frequency band and transmit power of the PED, the simulated *IPL* values were compared with the $IPL_{target}$ in terms of front door coupling for both spurious and intentional radiated emissions. The results indicate that under the worst-case scenario, PEDs with satellite communication functions may pose a risk of electromagnetic interference to the GPS L1 band. The *IPL* is up to 15 ± 3 dB lower than $IPL_{target}$, indicating the need for adequate electromagnetic shielding protection. Additionally, this study offers an assessment method to evaluate the coexistence of satellite communication-enabled mobile phones and aircraft GNSS systems (GPS L1).

## Figures and Tables

**Table 1:** Mesh setting for A320 cabin model.

Mesh Properties	Mesh Settings
Maximum cell	Cell per wavelength:
	near to model: 8; far from model: 8
	Cell per max model box edge:
	near to model: 20; far from model: 20
Minimum cell	Fraction of maximum cell near to model: 20
Mesh cells	41,987,280

Note: All simulations were performed on a PC platform with Intel(R) (Intel, Santa Clara, USA) Xeon(R) Gold 6242R CPU @ 3.10 GHz (two processors) with 512 GB RAM using the mesh settings detailed in Table 1.

**Table 2:** Statistical analysis of *IPL* values.

Influencing Factors	Min *IPL*	Max *IPL*	Avg *IPL*	SD	95% CI
PED at the left side of the 4th row of windows (1500–1600 MHz)	61.93	106.39	71.36	5.11	[71.04, 71.68]
PED at the center of the 4th row of windows (1500–1600 MHz)	62.16	105.58	71.81	5.63	[71.46, 72.16]
PED at the right side of the 4th row of windows (1500–1600 MHz)	61.75	95.73	71.60	5.40	[71.27, 71.94]
PED at the right side of the 40th row of windows (1500–1600 MHz)	64.30	97.84	75.86	5.22	[75.54, 76.18]
PED at the right side of the 4th row of windows (1980–2200 MHz)	69.07	108.25	79.88	5.60	[79.53, 80.22]
PED at the right side of the 4th row of windows (Vertical Polarization)	62.16	105.58	71.81	5.63	[71.46, 72.16]
PED at the right side of the 4th row of windows (Horizontal Polarization)	64.34	99.99	74.25	5.42	[73.91, 74.58]

## Data Availability

The data presented in this study are available on request from the corresponding author.
